# N-Dopant-Mediated Growth of Metal Oxide Nanoparticles on Carbon Nanotubes

**DOI:** 10.3390/nano11081882

**Published:** 2021-07-22

**Authors:** Jin Ah Lee, Won Jun Lee, Joonwon Lim, Sang Ouk Kim

**Affiliations:** 1 National Creative Research Initiative Center for Multi-Dimensional Directed Nanoscale Assembly, KAIST, Daejeon 34141, Korea; lja0508@naver.com (J.A.L.); wjlee@dankook.ac.kr (W.J.L.); sangouk.kim@kaist.ac.kr (S.O.K.); 2 Department of Materials Science and Engineering, KAIST, Daejeon 34141, Korea; 3 KAIST Institute for Nanocentury, KAIST, Daejeon 34141, Korea; 4 Department of Fiber System Engineering, Dankook University, Yongin 16890, Korea; 5 Department of Information Display, Kyung Hee University, Seoul 02447, Korea

**Keywords:** dopant, carbon nanotubes, metal oxides, nanoparticles, hybridization

## Abstract

Metal oxide nanoparticles supported on heteroatom-doped graphitic surfaces have been pursued for several decades for a wide spectrum of applications. Despite extensive research on functional metal oxide nanoparticle/doped carbon nanomaterial hybrids, the role of the heteroatom dopant in the hybridization process of doped carbon nanomaterials has been overlooked. Here, the direct growth of MnO_x_ and RuO_x_ nanoparticles in nitrogen (N)-doped sites of carbon nanotubes (NCNTs) is presented. The quaternary nitrogen (N_Q_) sites of CNTs actively participate in the nucleation and growth of the metal nanoparticles. The evenly distributed N_Q_ nucleation sites mediate the generation of uniformly dispersed <10 nm diameter MnO_x_ and RuO_x_ nanoparticles, directly decorated on NCNT surfaces. The electrochemical performance of the resultant hybridized materials was evaluated using cyclic voltammetry. This novel hybridization method using the dopant-mediated nucleation and growth of metal oxides suggests ways that heteroatom dopants can be utilized to optimize the structure, interface and corresponding properties of graphitic carbon-based hybrid materials.

## 1. Introduction

Substitutional heteroelement doping of graphitic carbon nanomaterials, including nitrogen (N), boron (B), sulfur (S) and fluorine (F), has been intensively investigated to control and optimize their structure and properties [1,2]. Such heterodopants can offer an opportunity for dramatic modifications in the electronic structure and chemical properties of graphitic carbon nanomaterials [3,4]. In addition, the modified surface properties of doped carbon nanomaterials have been widely exploited for nanocomposite synthesis. Controlled surface energy and enhanced chemical reactivity of the carbonous surface resulting from heteroatom doping facilitate a straightforward hybridization with various heteroelemental materials, including monomers [5], polymers [6], metals [7] and metal oxides [8]. In this regard, intensive research efforts have been made to develop efficient methods for the incorporation of heteroatoms into the hexagonal graphene lattice, such as covalent chemistry or thermal treatment with heteroatom sources [9,10].

Metal oxide nanoparticles have been explored for a wide spectrum of emerging applications, such as energy [11,12,13] and the environment [14,15]. In particular, metal oxide nanoparticles supported on electrically conductive carbon nanomaterials offer vast opportunities to achieve high-performance materials in energy conversion/storage applications, such as rechargeable batteries [16,17], supercapacitors [6] and water splitting [18,19]. Owing to the synergistic property of large effective surface area and high electrical conductivity, heteroatom-doped graphene and carbon nanotubes (CNTs) have been effectively exploited as a support material to maximize the electrochemical performance of functional metals [20,21] or metal oxide nanoparticles [22,23,24]. In contrast to the pristine graphitic surface, the doped graphitic surface allows the uniform formation of metal oxide nanoparticles. In addition, the modified graphitic surface by dopants provides suitable surface wettability, facilitating water- or polar-solvent-based wet synthetic processes [25]. However, although there has been extensive research exploiting the synergistic relationship between the heteroatom-doped graphitic surface and metal oxide nanoparticle decoration, the role of the heteroatom incorporated into the graphene network in the nucleation and growth of metal oxide nanoparticles is not clearly understood. Unveiling the hidden reaction mechanism of the uniform decoration of metal oxide nanoparticles on the heteroatom-doped graphitic surface is highly necessary for the rational design and synthesis of functional nanomaterials based on doped carbon nanomaterials.

We report a straightforward approach for the direct synthesis of MnO_x_ and RuO_x_ nanoparticles on a N-doped graphitic surface of vertically aligned NCNTs. The N dopant in NCNTs plays an important role in providing nucleation sites of metal oxide precursors at the early stage of the reaction, and it ultimately enables the highly uniform formation of metal oxide nanoparticles, evenly distributed over the entirety of NCNT surfaces. In addition, the substitutional N-doping process enhances the water wettability of the inherently hydrophobic graphitic surface and accordingly allows the metal oxide precursors to easily access the N nucleation sites. Due to the advantage of cooperative doping effects, the synthesized metal oxide nanoparticles exhibit a highly uniform size and are evenly distributed over NCNT surfaces. We also demonstrate the supercapacitor electrode performance of these idealized hybrid materials.

## 2. Materials and Methods

### 2.1. Materials

An asymmetric block copolymer, polystyrene-block-poly (methyl methacrylate) (PS-*b*-PMMA, molecular weight: PS/PMMA-46k/21k, P2400-SMMA), was purchased from Polymer Source Inc. (Dorval, QC, Canada). The iron source for electron beam evaporation (purity: 99.95%) was purchased from Thifine (Incheon, Korea). KMnO_4_ and RuCl_3_ for the synthesis of MnO_x_ and RuO_x_ nanoparticles were purchased from Sigma Aldrich.

### 2.2. Preparation of Nanopatterned Catalysts for NCNT Growth

A silicon wafer surface was neutralized by forming a thin film of PS-*r*-PMMA random copolymer brush by spin-coating. The purchased PS-*b*-PMMA block copolymer was spin-coated onto the reduced graphene oxide (rGO)/SiO_2_/Si wafer surface. After high-temperature annealing at 190 °C for self-assembly of PS-*b*-PMMA block copolymer, the substrates were UV irradiated and subsequently rinsed with acetic acid (purity: >99%) and water to remove the cylindrical PMMA cores from the cross-linked PS matrix. The substrate was further treated in oxygen plasma for 20 s in order to remove the remaining cylindrical cores. The Fe catalyst film was deposited over the block copolymer template. After the deposition process, the remaining PS nanoporous template was lifted off by sonication in toluene (purity: >99.5). All solvents were used as received.

### 2.3. Growth of Vertically Aligned NCNTs

NCNT growth was carried out on Fe catalyst-deposited substrates by plasma enhanced chemical vapor deposition (PECVD) method. The substrate was heated to 700 °C under a mixture of hydrogen (H_2_, 60 sccm, 99.99% purity) and ammonia (NH_3_, 40 sccm, 99.999% purity) gases (chamber pressure: 0.4 torr) for 3 min to form catalyst particles with an isotropic shape. The chamber pressure was increased to 5 torr, and DC plasma was activated with an anode DC voltage of 470 V with respect to the ground (0 V). Slowly streaming the acetylene (C_2_H_2_, 99.5% purity) source gas at a flow rate of 25 sccm for 1 min led to a dense and vertically aligned NCNT forest.

### 2.4. Synthesis of MnO_x_ and RuO_x_ Nanoparticles Supported on NCNTs

Prior to hybridization, NCNTs were immersed in ethanol for 1 min to enhance wettability. The decoration of MnO_x_ nanoparticles on the surface of NCNTs was performed by placing the NCNTs in 10 mM and 100 mM KMnO_4_ aqueous solution at 60 °C with mild stirring. For RuO_x_ nanoparticle decoration, NCNTs were immersed in 10 mM RuCl_3_ aqueous solution for 20 min. A 0.1 M NaOH solution was added dropwise at 70 °C after the desired reaction time, the samples were thoroughly washed with deionized water to remove unreacted residual metal oxide precursors. The as-synthesized MnO_x_/NCNT and RuO_x_/NCNT were dried at 400 °C and 200 °C, respectively, for 10 min in a vacuum oven. The vacuum-drying process helped to reduce undesirable agglomeration between metal oxide nanoparticle-decorated NCNTs. 

### 2.5. Characterization

The morphology of the prepared metal oxide–decorated NCNTs was characterized by a field-emission scanning electron microscope (SEM) (Hitachi S-4800; Hitachi, Tokyo, Japan) and a transmission electron microscope (TEM) (Tecnai F20; FEI Company, Hillsboro, OR, USA). Chemical characterization of the sample was conducted using X-ray photoelectron spectroscopy (XPS) (K-alpha; Thermo Scientific, Waltham, MA, USA). All electrochemical experiments were performed using a three-electrode cell configuration. A mesh-type platinum and a standard calomel electrode (SCE) were used as the counter electrode and the reference electrode, respectively. Cyclic voltammetry tests were used to evaluate the electrochemical performance of the electrodes for supercapacitor applications with Bio-Logic (SP-200, Seyssinet-Pariset, France) in 1 M H_2_SO_4_ electrolyte. Supercapacitor electrodes were prepared by the transfer of CNT hybrids from the SiO_2_/Si wafer to fluorine-doped tin oxide (FTO) glass used as a working electrode.

## 3. Results and Discussion

**Figure 1:**
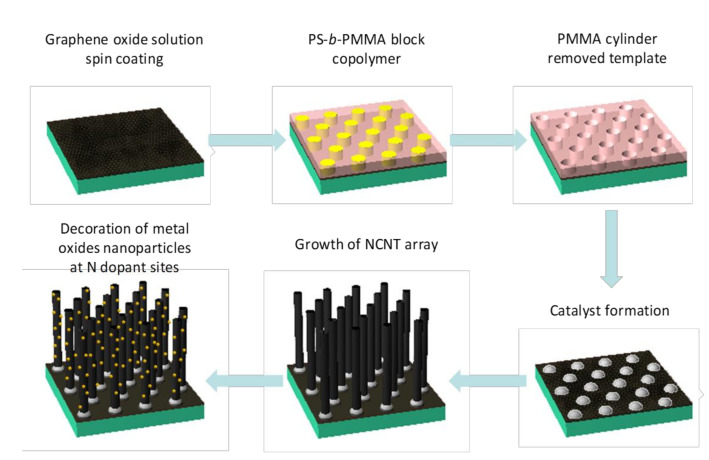
A schematic diagram for the procedure of metal oxide nanoparticle decoration on NCNTs.

Figure 1 illustrates a schematic diagram of the procedure for the synthesis of MnO_x_ and RuO_x_ nanoparticles directly supported on each NCNT strand. Highly aligned vertical NCNTs were grown from iron (Fe) catalysts by using PECVD in a NH_3_ environment, which was used as a chemical source for the N-doping of CNTs. The diameter of each NCNT strand and the distance between NCNTs were precisely controlled to ~20 nm and ~50 nm, respectively, by controlling the size and the location of Fe catalysts using a nano-template made of self-assembled cylindrical PS-*b*-PMMA block copolymer (BCP) (Figure S1). The well-controlled growth of NCNTs is essential for a systematic study on the role of the N dopant in the metal nanoparticle synthesis because it minimizes unwanted influencing factors, such as agglomeration of CNTs and poor access of precursors to the NCNT surfaces during the hybridization process. The grown NCNTs were immersed in a precursor solution of KMnO_4_ or RuCl_3_ for the synthesis of MnO_x_ or RuO_x_ nanoparticles, respectively [26,27]. After the desired reaction time, samples were washed with deionized water to remove unreacted residual precursors from the produced metal oxide nanoparticle and NCNT hybrids. Subsequently, the as-synthesized MnO_x_/NCNT and RuO_x_/NCNT were annealed at 400 °C and 200 °C, respectively, in an Ar environment for 10 min. Owing to the modified surface properties of NCNTs due to the formation of metal oxide nanoparticles, the vertical alignment of the grown NCNTs remained intact after the drying process without an easily observed aggregation phenomenon of graphitic nanomaterials, originating from a capillary effect. 

**Figure 2:**
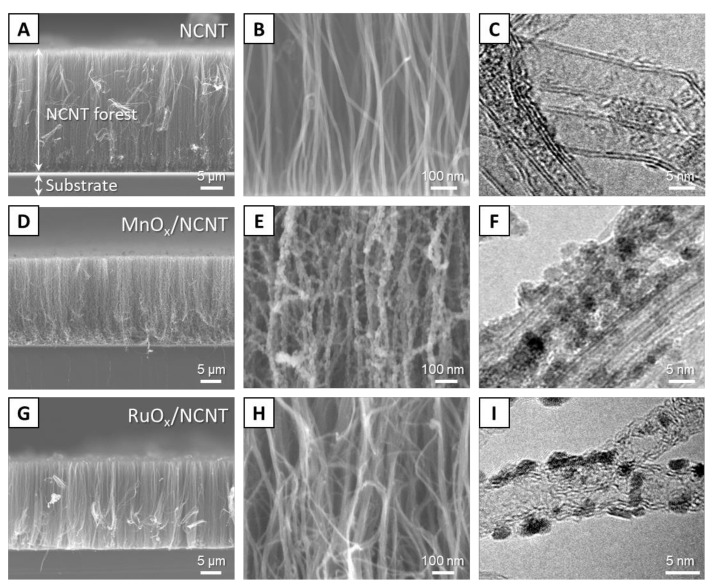
SEM and TEM analysis of NCNT (**A**–**C**), MnO_x_/NCNT (**D**–**F**) and RuO_x_/NCNT (**G**–**I**).

The morphology of NCNTs and metal oxide hybrids/NCNT was observed by scanning electron microscopy (SEM) and transmission electron microscopy (TEM). As shown in Figure 2A,B, the grown NCNTs from the controlled Fe catalysts have well-aligned vertical alignment and are properly separated from each other without agglomerated or bundled structures. The height of the NCNT forest was around 25 μm. As shown in the high-magnification TEM image in Figure 2C, the diameter of the grown NCNTs is highly uniform at around 10 nm, which results from the precise size control of Fe catalysts. Figure 2D,E show SEM images of MnO_x_/NCNT hybrids. Even after the water-based hybridization process, the initial vertical alignment was well-preserved due to the modified surface energy of NCNTs by the conformal coating of MnO_x_ nanoparticles on the surface of each NCNT strand. Interestingly, the MnO_x_ nanoparticles directly synthesized on the N-doped graphitic surface have a spherical shape with a highly uniform diameter of less than 10 nm (Figure 2F). They are distinct from flake-like or rod-like MnO_x_ nanoparticles, generally synthesized on pristine CNTs, as reported in many previous studies [28,29,30]. The conformal and uniform formation of MnO_x_ nanoparticles on NCNTs can be elucidated with the even distribution of N dopants over the whole surface and the well-aligned separated structures of the grown NCNTs, which prevent non-uniform nucleation or hidden surfaces resulting from agglomeration. Similar morphological features were observed in the case of hybridization between RuO_x_ and NCNTs, as shown in Figure 2G–I. 

**Figure 3:**
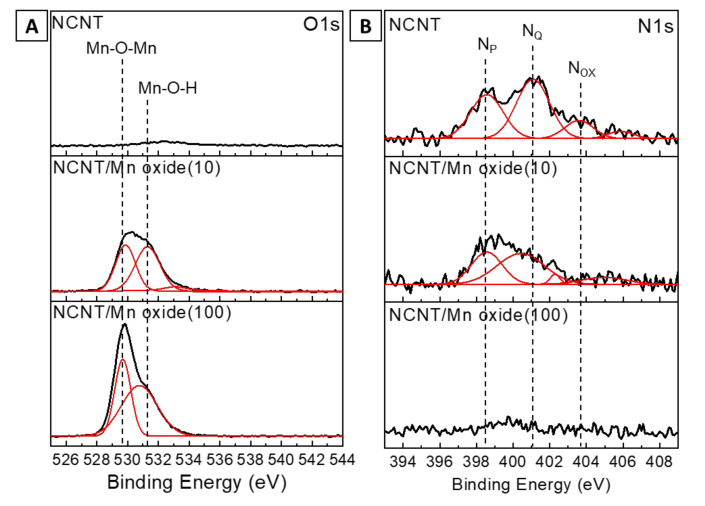
XPS analysis of NCNTs and MnO_x_/NCNT hybrids. (**A**) O1s XPS spectra. (**B**) N1s XPS spectra. The black and red lines show as-received XPS results and deconvoluted peaks, respectively. The number in parentheses indicates the molar concentration of KMnO_4_ in the Mn precursor solution used for hybridization process.

X-ray photoelectron spectroscopy (XPS) was carried out to analyze the chemical structure of MnO_x_/NCNT hybrids. The grown NCNTs contain N contents of ~2.8 at% with respect to C content, as shown in Figure S2. The Mn 2p, O1s and N1s XPS spectra of NCNTs and MnO_x_/NCNT are presented in Figure 3. The number in parentheses in Figure 3 indicates the molar concentration of KMnO_4_ in the Mn precursor solution used for the hybridization process. In the Mn 2p spectrum of MnO_x_ (Figure S3), the two peaks located at 641.3 eV and 653.0 eV have an energy separation of 11.7 eV, indicating that the oxidation state of Mn is +4 [31]. In other words, the synthesized MnO_x_ nanoparticles are mainly composed of MnO_2_. The O1s spectra of NCNTs, NCNT/MnO_x_ (10) and NCNT/MnO_x_ (100) are deconvoluted into two main peaks of characteristic oxygen bonds, which are related to the Mn–O–Mn bond (both 529.7 eV) and the Mn–O–H bond (both 531.3 eV), respectively (Figure 3B). Notably, MnO_x_/NCNT (100) synthesized in 100 mM KMnO_4_ solution shows a larger peak at 529.7 eV for the Mn–O–Mn bond compared to that of MnO_x_/NCNT (10), indicating the appropriate growth of MnO_x_ nanoparticles in the given reaction time. Figure 3B compares the N1s XPS spectra of the three samples. The deconvoluted spectra of bare NCNTs clearly show the presence of pyridinic nitrogen (N_P_, 398.4 eV), quaternary nitrogen (N_Q_, 401.1 eV) and nitrogen oxide (N_ox_, 403.8 eV) [32]. The chemical configuration of N_P_ and N_Q_ are illustrated in Figure S4. The N1s XPS spectra of MnO_x_/NCNT hybrids definitely show a weakened peak intensity. It is noteworthy that while the peak intensity for N_P_ of MnO_x_/NCNT (10) is slightly decreased, the peak intensity for N_Q_ is drastically reduced with respect to that of bare NCNTs. Similarly, an apparent reduction in the peak intensity for N_Q_ is observed in the N1s XPS spectra of RuO_x_/NCNT (10) (Figure S5). This indicates that the N_Q_ moiety provides an energetically favorable nucleation site for the formation of MnO_x_ nanoparticles. When the concentration of the precursor solution was increased from 10 mM to 100 mM, N1s XPS peaks became negligible, indicating the conformal and compact formation of MnO_x_ nanoparticles on the NCNT surface.

**Figure 4:**
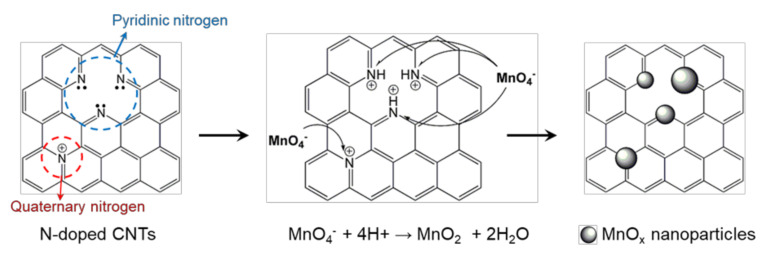
Schematic diagram of possible nucleation mechanism of MnO_x_ nanoparticles at quaternary N (N_Q_) and pyridinic N (N_P_).

Figure 4 presents the schematic diagram of a possible nucleation mechanism of MnO_x_ nanoparticles from MnO_4_^−^ ions at N_Q_ and N_p_ dopant sites. The N_Q_ dopant, which substitutes a carbon atom in the hexagonal lattice of the graphene plane, is known to have a partially positive charge (+δ), which it acquires by donating electrons to the π-conjugated system of graphene [33]. The positively charged N_Q_ dopant site can electrostatically interact with MnO_4_^−^ ions in the precursor solution. Thus, the negatively charged precursor ions prefer the attractive N_Q_ sites over other atomic sites to stably form a seed for the growth of MnO_x_ nanoparticles. The positively charged N_P_ dopant sites, which are readily formed by protonation in an acidic solution, can act as nucleation sites. However, the contribution of protonated N_P_ dopant sites to uniform MnO_x_ nucleation could be less predominant due to the use of a neutral KMnO_4_ solution in this work. The uniform formation of RuO_x_ nanoparticles on the NCNT surface can be similarly rationalized (Figure S6). Hence, the evenly distributed and positively charged N dopant sites enable the highly uniform and stable formation of metal oxide on NCNTs without any additional treatments or adhesion layers. 

**Figure 5:**
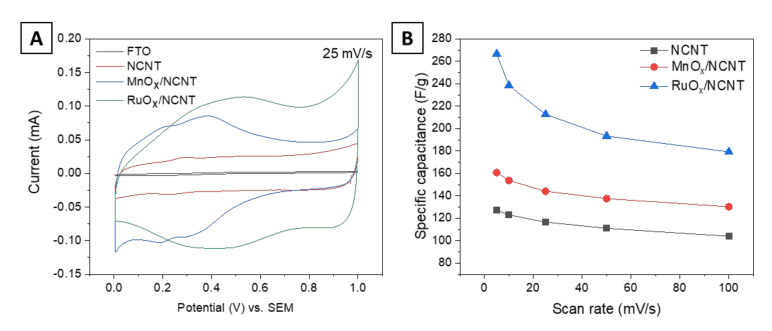
Electrochemical characterization. (**A**) Cyclic voltammograms of NCNTs, MnO_x_/NCNT and RuO_x_/NCNT at a scan rate of 25 mV/s. (**B**) Gravimetric specific capacitance vs. scan rate for NCNTs, MnO_x_/NCNT and RuO_x_/NCNT.

Owing to the synergistic effect of the highly active electrochemical redox behavior of MnO_x_ and RuO_x_ and highly conductive NCNT core, MnO_x_/NCNT and RuO_x_/NCNT are expected to be excellent candidates for a supercapacitor electrode. The electrochemical performance of supercapacitor electrodes was evaluated using cyclic voltammetry (CV) at room temperature. A typical three-electrode configuration was used in 1 M H_2_SO_4_ electrolyte. Figure S7A–C show the CV curves of NCNT- MnO_x_/NCNT- and RuO_x_/NCNT-based supercapacitors, respectively, with various scan rates in a potential range of 0–1.0 V (vs. saturated calomel electrode (SCE)). The voltammograms of the NCNTs in Figure S7A show a nearly rectangular shape, indicating charge storage by the adsorption–desorption mechanism forming electrical double layers (EDLs) [34]. In contrast, MnO_x_/NCNT and RuO_x_/NCNT exhibit pseudo-capacitive behaviors, represented by Faradaic redox peaks in the voltammograms (Figure S7B,C) [35]. Owing to the additional energy storage by the redox reactions on the surfaces of metal oxide nanoparticles [36], MnO_x_/NCNT and RuO_x_/NCNT show an enhanced charge storage capacity of 141.1 F/g and 208.2 F/g at a scan rate of 25 mV/s, respectively, compared to 116.6 F/g for the NCNT electrode (Figure 5A). However, the redox charge transfer-based energy storage mechanism of MnO_x_/NCNT and RuO_x_/NCNT electrodes results in low rate capability compared to the NCNT electrode, as shown in Figure 5B. The specific capacitance retention of NCNTs, MnO_x_/NCNT and RuO_x_/NCNT after charge–discharge at 100 mV/s was determined to be 82%, 75% and 67% of those at 5 mV/s, respectively.

## 4. Conclusions

In summary, we demonstrated the straightforward synthesis of metal oxide nanoparticles, which were directly decorated on vertically aligned NCNTs by exploiting N dopant moieties as stable nucleation sites. Taking advantage of precisely controlled NCNT arrays based on Fe catalyst nanopatterns using PS-*b*-PMMA nano-template, the effect of N dopants in NCNTs was investigated in detail. Positively charged N_Q_ dopant sites, evenly distributed over NCNT walls, act as nucleation sites for the stable and uniform formation of MnO_x_ and RuO_x_ by attracting negatively charged precursor ions in aqueous solution. The uniformly coated MnO_x_ and RuO_x_ nanoparticles on the NCNT surface remarkably improved the electrochemical energy storage capability to 141.1 F/g and 208.2 F/g, respectively, compared to 116.6 F/g for NCNTs. The presented synthetic strategy using the dopant-mediated nucleation of metal oxides suggests ways that heteroatom dopants can be utilized to optimize the structure, interface and corresponding properties of graphitic carbon-based hybrid materials.

## Data Availability

Not applicable.

## Supplementary Materials

The following are available online at https://www.mdpi.com/article/10.3390/nano11081882/s1, Figure S1: Preparation of Fe catalysts using PS-*b*-PMMA nano-templates; Figure S2: Survey scan and N1s XPS spectra of NCNTs; Figure S3: Chemical configuration of nitrogen dopants in NCNT; Figure S4: XPS analysis of RuO_x_/NCNT hybrids; Figure S5: Schematic diagram of the nucleation of RuO_x_ nanoparticles at N_Q_ or N_P_ dopant sites in NCNT.
