# Supplementary material for: N-Dopant-Mediated Growth of Metal Oxide Nanoparticles on Carbon Nanotubes
Source: Nanomaterials (Basel). 2021 Jul 22;11(8):1882. doi: 10.3390/nano11081882 (PMC8400905; doi:10.3390/nano11081882)
Supplement: Supplementary file 1 [file nanomaterials-11-01882-s001.zip › nanomaterials-1291505-supplementary.pdf]

Supporting Information

N-Dopant-Mediated Growth of Metal Oxide Nanoparticles on Carbon Nanotubes

Jin Ah Lee [1,2,3], Won Jun Lee [1,2,3,4], Joonwon Lim [1,2,3,5,*] and Sang Ouk Kim [1,2,3]

[1] National Creative Research Initiative Center for Multi-Dimensional Directed Nanoscale Assembly, KAIST, Daejeon 34141, Korea; lja0508@naver.com (J.A.L.); wjlee@dankook.ac.kr (W.J.L.); sangouk.kim@kaist.ac.kr (S.O.K.)
[2] Department of Materials Science and Engineering, KAIST, Daejeon 34141, Korea
[3] KAIST Institute for Nanocentury, KAIST, Daejeon 34141, Korea
[4] Department of Fiber System Engineering, Dankook University, Yongin-si 16890, Korea
[5] Department of Information Display, Kyung Hee University, Seoul 02447, Korea
* Correspondence: joonwon.lim@khu.ac.kr

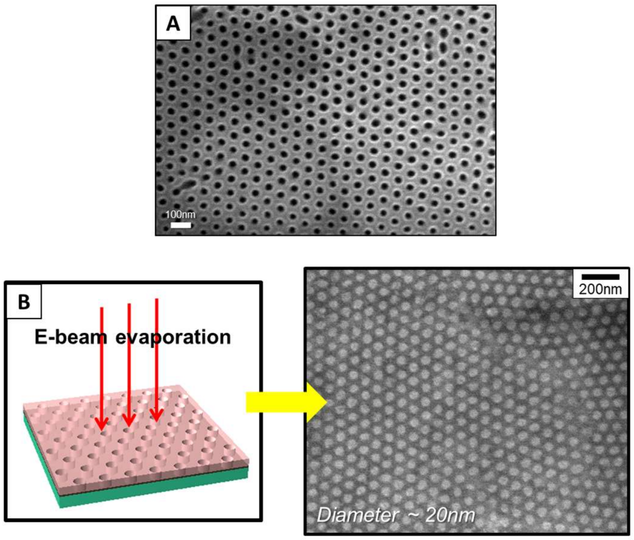

Figure S1. Preparation of Fe catalysts using PS-*b*-PMMA nanotemplates (**A**) PS-*b*-PMMA cylinder type block copolymer template after the removal of PMMA core. (**B**) Hexagonally packed Fe catalyst arrays prepared by E-beam evaporation with PS-*b*-PMMA nanotemplate in (**A**).

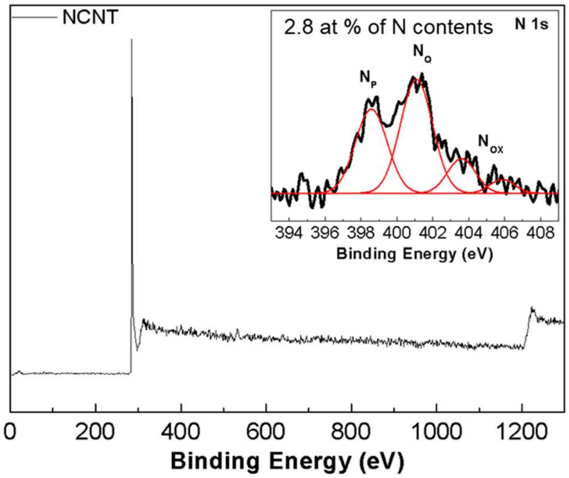

Figure S2. Survey san and N 1s XPS spectra of NCNTs

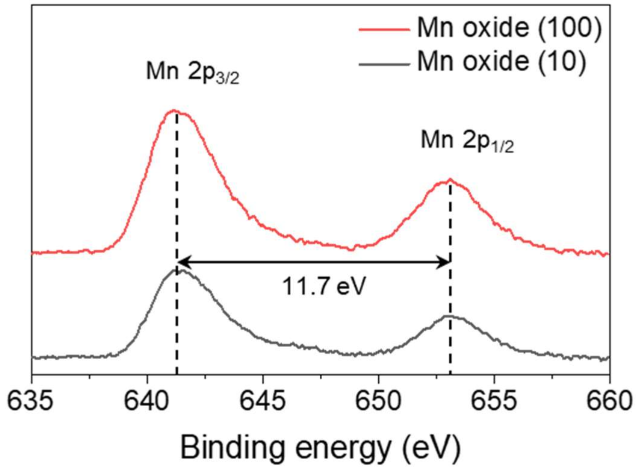

Figure S3. Mn 2p XPS Spectrum of Mn oxide (100)/NCNT and Mn oxide (10)/NCNT. The number in parentheses indicates the molar concentration of $KMnO_4$ in a Mn precursor solution used for hybridization process.

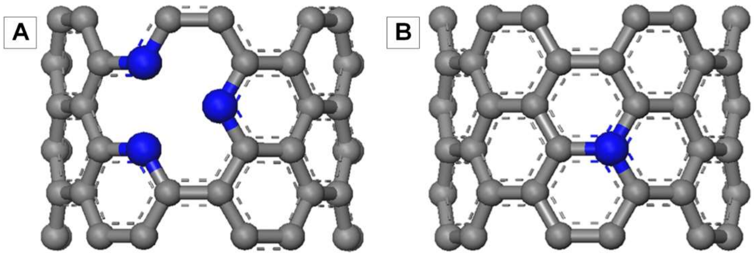

Figure S4. Chemical configuration of nitrogen dopants in NCNT. (**A**) Pyridinic nitrogen (N_P), (**B**) Quaternary nitrogen (N_Q)

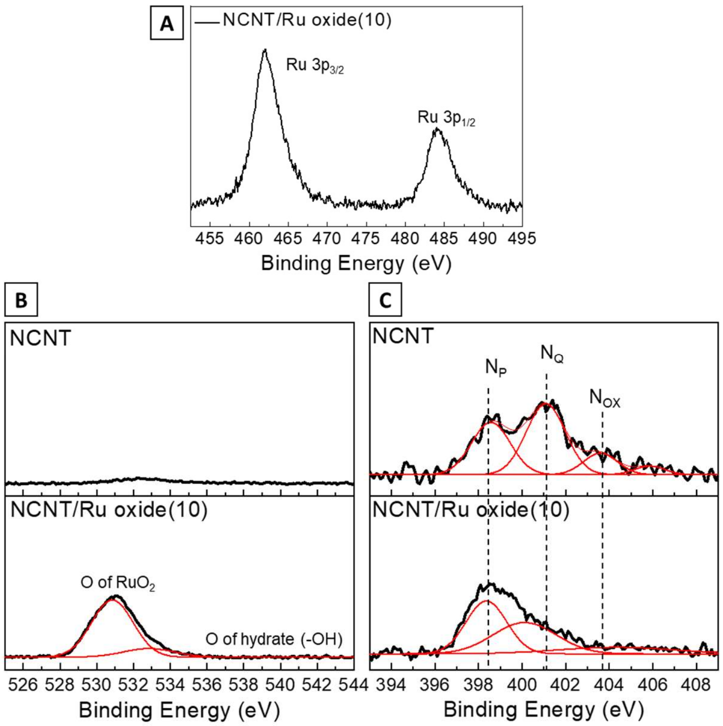

Figure S5. XPS analysis of RuO_x/NCNT hybrids.

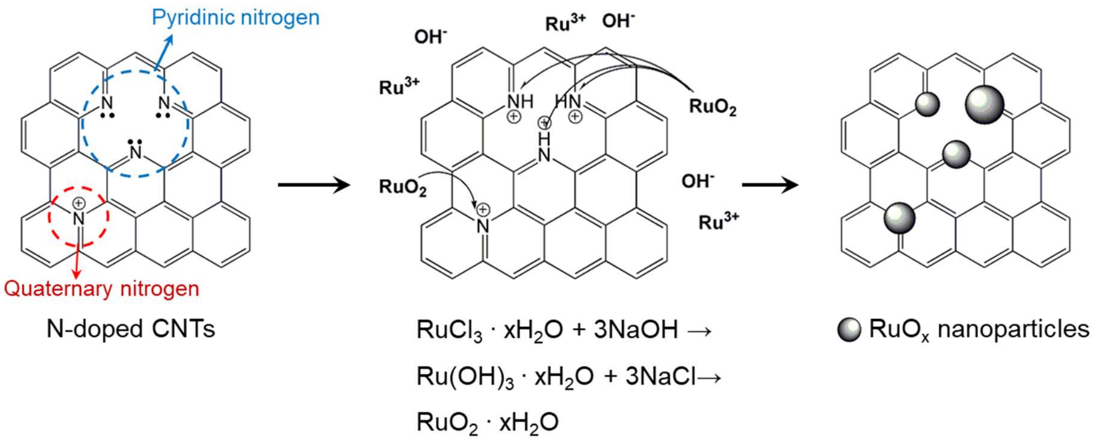

Figure S6. Schematic diagram of the nucleation of RuO_x nanoparticles at N_Q or N_P dopant sites in NCNT

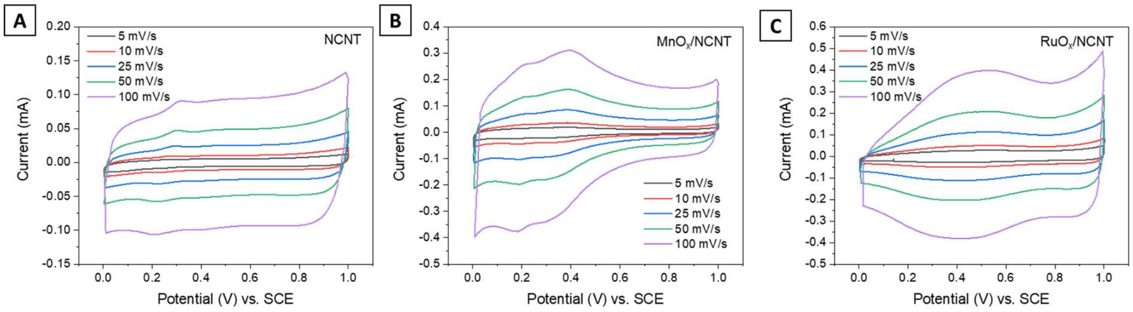

Figure S7. Electrochemical characterization. (**A–C**) Cyclic voltammograms at various scan rates of NCNT, MnO_x/NCNT and RuO_x/NCNT. The cathodic peak at 0.2 V vs. SCE in (**A**) originates from the redox reaction of residual Fe elements used as a catalyst for NCNT growth.
